# Impact of aortic root repair or replacement in severe destructive aortic valve endocarditis with paravalvular abscesses on long-term survival

**DOI:** 10.1093/icvts/ivab330

**Published:** 2021-12-06

**Authors:** Can Gollmann-Tepeköylü, Hannes Abfalterer, Leo Pölzl, Ludwig Müller, Michael Grimm, Johannes Holfeld, Nikolaos Bonaros, Katie Bates, Hanno Ulmer, Elfriede Ruttmann

**Affiliations:** 1 Department of Cardiac Surgery, Medical University of Innsbruck, Innsbruck, Austria; 2 Department of Clinical and Functional Anatomy, Medical University of Innsbruck, Innsbruck, Austria; 3 Department of Medical Statistics, Informatics, and Health Economics, Innsbruck Medical University, Innsbruck, Austria

**Keywords:** Infective endocarditis, Destructive aortic valve endocarditis, Aortic root replacement, Aortic root repair

## Abstract

**OBJECTIVES:**

Surgical treatment of destructive infective endocarditis consists of extensive debridement followed by root repair or replacement. However, it remains unknown whether 1 is superior to the other. We aimed to analyse whether long-term results were better after root repair or replacement in patients with root endocarditis.

**METHODS:**

A total of 148 consecutive patients with root endocarditis treated with surgery from 1997 to 2020 at our department were included. Patients were divided into 2 groups: aortic root repair (*n* = 85) or root replacement using xenografts or homografts (*n* = 63).

**RESULTS:**

Patients receiving aortic root repair showed significantly better long-term survival compared to patients receiving aortic root replacement (log-rank: *P* = 0.037). There was no difference in terms of freedom from valvular reoperations among both treatment groups (log-rank: *P* = 0.58). Patients with aortic root repair showed higher freedom from recurrent endocarditis compared to patients with aortic root replacement (log-rank: *P* = 0.022). Patients with aortic root repair exhibited higher event-free survival (defined as a combination end point of freedom from death, valvular reoperation or recurrent endocarditis) compared to patients receiving aortic root replacement (log-rank: *P* = 0.022). Age increased the risk of mortality with 1.7% per year. Multi-variable adjusted statistical analysis revealed improved long-term event-free survival after aortic root repair (hazards ratio: 0.57, 95% confidence interval: 0.39–0.95; *P* = 0.031).

**CONCLUSIONS:**

Aortic root repair and replacement are feasible options for the surgical treatment of root endocarditis and are complementary methods, depending on the extent of infection. Patients with less advanced infection have a more favourable prognosis.

**Clinical trial registration:**

UN4232 382/3.1 (retrospective study).

**Figure:**
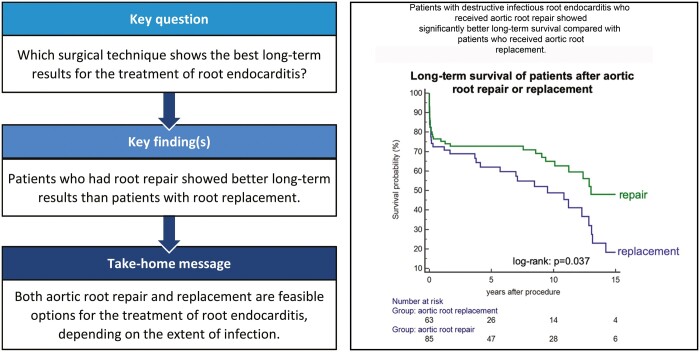

## INTRODUCTION

Infective endocarditis (IE) remains a deadly disease with an incidence of 30 to 100 episodes per million patient-years [1]. Despite optimal medical therapy and aggressive surgical repair, more than a third of the patients die within the first year of diagnosis [2]. The management includes optimal antibiotic therapy according to the underlying microbial infection and antibiogram [3]. In case of complicated IE, early surgical treatment is indicated [3].

Surgical treatment of root endocarditis requires extensive debridement of destroyed tissue with subsequent annular repair and valvular replacement or root replacement [4]. For this purpose, various approaches have been described over the last years. The use of homografts has been reported successfully in 103 consecutive patients with recurrence of IE in only 3.8% [5]. Resistance to infection is attributed to the preservation treatment of homografts, possibly by the induction of the immunomodulator indoleamine 2,3-dioxygenase [6]. However, availability of homografts is limited and early calcification of conduits has been reported [7].

Another valuable choice for root replacement in endocarditis is the Freestyle prosthesis [8]. The xenograft with unlimited availability is attributed with lower calcification properties than homografts and, thus, fewer rates of progressive aortic valve dysfunction [9]. However, as many of patients with root endocarditis are <60 years, the Freestyle prosthesis contains the disadvantage of progressive prosthesis degeneration requiring complex reoperations in younger patients. For this population, the annular reconstruction upon abscess resection with subsequent (mechanical) valve replacement might be an alternative treatment option [10].

The current ESC endocarditis guidelines remain vague regarding the choice of prosthesis in patients with root endocarditis [3], as there are still very limited data on this complex patient population. Thus, there is an urgent need for larger case series to define the treatment of choice for patients with root endocarditis.

## PATIENTS AND METHODS

### Ethics statement

The trial complies with the Declaration of Helsinki and permission to perform this study was obtained from the Innsbruck Medical University Institutional Review Board [UN4232 382/3.1 (retrospective study)].

### Study design

Data were derived from a consecutive series of patients with left-sided IE undergoing cardiac surgery during the active phase of infection from 1997 to 2020 at the University Hospital Innsbruck.

A total of 490 patients were operated for active IE during this period. Among them, 286 patients suffered from aortic valve endocarditis (58.4%) and 148 patients (51.7%) had additional paravalvular root abscesses. These 148 patients with aortic root abscesses served as the study population for this present investigation.

Patients were divided into 2 groups according to the operative strategy, namely first aortic root repair with patch reconstruction of the aortic root and conventional implantation of an aortic prosthesis and second aortic full root replacement using xenografts or homografts.

IE was diagnosed according to the modified Duke criteria based on clinical signs, blood culture, histologic examination, and transoesophageal echocardiography. Indication for surgery in these patients was based on the transoesophageal echocardiography results of root abscess or intracardiac fistula formation. Severe excavating aortic valve endocarditis was defined by intraoperative findings of acute necrotizing endocarditis with vegetations and partial or total destruction of the annulus and left ventricular outflow tract (LVOT).

Technical success of surgery (successful reconstruction of the aortic root and LVOT), perioperative complications (e.g. mortality and early recurrence of IE) and long-term outcome regarding survival, late prosthetic valve endocarditis and late prosthetic dysfunction due to degeneration needing reoperation were analysed. In accordance with the STS guidelines, perioperative mortality was defined as mortality within 30 days or during initial hospital stay. Late mortality was defined as death thereafter. The median follow-up time of the entire cohort was 9.0 years (range 3 months to 23 years).

Operations were considered as emergent if performed within 24 h after diagnosis for haemodynamic instability, and urgent if performed during the index hospital admission.

All patients had pre- and intraoperative transoesophageal echocardiography to measure vegetation size, severity of valvular dysfunction or paravalvular abscess formation. Indications for early surgery were haemodynamic deterioration, high embolic risk (large vegetation size), para-valvular abscesses/fistulation or non-controllable sepsis despite appropriate antibiotic treatment.

### Surgical technique

All surgical procedures were performed with the use of cardiopulmonary bypass (CPB) under mild hypothermia (32–35°C) and full anticoagulation. Heparinization was frequently monitored during extracorporeal circulation and was maintained by activated clotting times >480 s to avoid clotting. Haematocrit levels were kept between 26% and 30% by priming of the CPB or by adding erythrozytes to the CPB circuit.

Surgery included the complete transection of the aorta, which was performed after preliminary evaluation of the extent and severity of the disease, and radical resection of all infected and necrotic tissue was done without consideration of the extent of the created defect, and later reconstruction or creation of heart block. Typically, the full extent of the destructive process became evident only after radical debridement. Specimens of the valve and necrotic material were sent for bacteriologic and histopathologic investigation.

Reconstruction of the LVOT and aortic root was accomplished by means of a Freestyle xenograft aortic root (in the majority of cases) in the aortic root replacement group and bovine pericardial patches for LVOT and aortic root in the aortic root repair group. The decision to repair or replace was made by the treating surgeon.

In case of aortic root replacement, a full root replacement with reimplantation of the coronary ostia using the button technique was performed. The xenograft was sutured to the LVOT with interrupted or running 4–0 Prolene sutures or a combination of both depending on the surgeon’s preference and the individual situation. Interrupted sutures were usually tied over a strip of pericardium to prevent leakage. A 5–0 Prolene was used for implantation of the coronary buttons. For the distal anastomosis between the xenograft/homograft and the ascending aorta, a 4–0 Prolene running suture was used which was buttressed by a strip of pericardium to prevent leakage from the Xeno/homograft. Intracardiac defects were also closed with pericardium. For this, 0.5% glutaraldehyde fixed autologous or bovine pericardium was used. The same material was used to repair defects of the mitral valve if present.

In case of aortic root repair, bovine pericardium was used to patch the LVOT and the aortic root defect and was sutured with running 4–0 Prolene into the aortic root. The decision on which valve prosthesis, either mechanical or biological, should be selected, was dependent on the age of the patient and the judgement of the attending surgeon. The perioperative antibiotic therapy was based on blood and tissue cultures and was continued for at least 6 weeks or even longer in specific cases.

### Follow-up

All patients were evaluated by the endocarditis heart team of our hospital preoperatively in order to evaluate indication and timing of surgery and operability of the patient. All survivors were followed up frequently by experienced cardiologists and neurologists and underwent clinical control- and echocardiographic examinations. Prosthetic dysfunction was evaluated thoroughly by experienced cardiologists via echocardiography in all 2D transthoracic views evaluating cusp morphology and haemodynamic performance via doppler echocardiography as recommended previously [1]. Autopsies were performed among all non-survivors to evaluate the cause of death and neurologic complications, such as secondary cerebral bleeding.

The closing interval for this study was between January 2020 and April 2020. Follow-up was 100% complete.

### Statistical analysis

Data are presented as mean ± SD for continuous variables and absolute numbers as well as percentages for categorical variables. The 2 patient groups (either aortic root replacement or aortic root repair) were compared for differences in demographic patient characteristics, surgical variables and perioperative outcomes. Comparisons between the 2 groups were performed for categorical variables with the chi-squared or Fisheŕs exact test, as appropriate. Continuous variables were compared by Student’s *t*-test or the Mann–Whitney *U*-test. Long-term survival, freedom from recurrent endocarditis and freedom from valvular reoperation between the 2 groups were assessed using Kaplan–Meier survival curves together with log-rank testing. For this purpose a combination end point of these outcome measures was defined to indicate ‘event-free survival’. Age-adjusted hazard ratios were calculated using Cox proportional-hazards regression analysis, respectively, to calculate the relative risks for long-term event-free survival with α = 0.05. Data documentation and statistical analysis were performed using SPSS 24.0 (IBM Corp.).

### Data availability statement

The data underlying this article will be shared on reasonable request to the corresponding author.

## RESULTS

The median follow-up time of the entire cohort was 9.0 years (range 3 months to 23 years). A detailed patient description of patients receiving either aortic root repair or aortic root replacement for destructive aortic valve endocarditis with perivalvular abscesses is displayed in Table 1. Eighty-five patients received aortic root repair and 63 patients received aortic root replacement.

**Table 1:** Characteristics of patients receiving either aortic root repair or aortic root replacement for destructive aortic valve endocarditis with perivalvular abscesses

	Aortic root repair	Aortic root replacement	*P*-value
	*n* = 85 patients	*n* = 63 patients	
Age (years), mean ± SD	56.3 ± 15.5	60.9 ± 14.3	0.07
Male gender, *n* (%)	66 (77.6)	44 (69.8)	0.28
NYHA stage prior to surgery, *n* (%)			
NYHA I	0 (0)	1 (1.6)	
NYHA II	19 (22.4)	9 (14.3)	
NYHA III	45 (52.9)	36 (57.1)	
NYHA IV	21 (24.7)	17 (27.0)	0.43
Previous cardiac decompensation, *n* (%)	57 (67.1)	40 (63.5)	0.65
Body mass index (kg/m^2^), mean ± SD	25.3 ± 4.6	24.4 ± 4.0	0.22
Obesity, *n* (%)	15 (17.6)	6 (9.5)	0.16
Arterial hypertension, *n* (%)	27 (32.1)	25 (40.3)	0.31
Hypercholesterolaemia, *n* (%)	24 (28.6)	24 (38.7)	0.20
Diabetes, *n* (%)	11 (13.1)	6 (9.5)	0.50
Peripheral vascular disease, *n* (%)	12 (14.1)	2 (3.3)	0.07
Chronic obstructive pulmonary disease, *n* (%)	24 (29.3)	15 (24.2)	0.50
Renal insufficiency (GFR < 30), *n* (%)	35 (41.2)	22 (34.9)	0.40
Left ventricular ejection fraction (%), mean ± SD	48.6 ± 12.2	46.4 ± 12.0	0.29
Cerebral stroke prior to surgery, *n* (%)	32 (37.6)	15 (23.8)	0.07
Spleen infarct/abscess, *n* (%)	14 (16.5)	5 (7.9)	0.13
Kidney infarct/abscess, *n* (%)	11 (12.9)	3 (4.8)	0.15
Liver infarct/abscess, *n* (%)	3 (3.5)	0 (0.0)	0.26
Prosthetic valve endocarditis, *n* (%)	21 (24.7)	40 (63.5)	<0.001
Double valve endocarditis, *n* (%)	23 (27.1)	8 (12.7)	0.03
Staphylococcal endocarditis, *n* (%)	52 (63.4)	39 (62.9)	0.95
Causative microorganism, *n* (%)			
No causative organism detected	9 (10.6)	7 (11.1)	
*Staphylococcus*	51 (60.0)	39 (61.9)	
*Streptococcus*	14 (16.5)	4 (6.3)	
*Enterococcus*	6 (7.1)	9 (14.3)	
Staph + *Streptococcus*	1 (1.2)	0 (0.0)	
Others	3 (3.5)	4 (6.3)	
*Aspergillus*	1 (1.2)	0 (0.0)	0.32
Additional CABG, *n* (%)	10 (11.8)	16 (25.4)	0.03
Duration of antibiotic treatment prior to surgery (days), mean ± SD	7.0 ± 6.7	7.0 ± 5.4	0.96
Latency between beginning of antibiotic treatment and surgery, *n* (%)			
0–3 days	25 (29.4)	21 (33.3)	
4–7 days	32 (37.6)	22 (34.9)	
>7 days	28 (32.9)	20 (31.7)	0.87
Primary indication for surgery, *n* (%)			
Haemodynamic deterioration	21 (24.7)	17 (27.0)	
Risk of embolism	49 (55.3)	25 (39.7)	
Uncontrollable sepsis	15 (17.6)	21 (33.3)	0.24
Additive EuroScore (points), mean ± SD	13.0 ± 7.8	15.4 ± 9.3	0.08
Hospital mortality, *n* (%)	16 (18.8)	19 (30.2)	0.11

SD: standard deviation.

Patient receiving aortic root repair was slightly younger than patients receiving aortic root replacement (56.3 ± 15.5 vs 60.9 ± 14.3 years, *P* = 0.07). Moreover, patients with aortic root repair showed higher rates of peripheral vascular disease (14.1% vs 3.3%, *P* = 0.07). In addition, cerebral stroke prior to surgery was more present in the aortic root repair group (37.6% vs 23.8%, *P* = 0.07). Patients receiving aortic root repair were more likely to have double valve endocarditis compared to patients with aortic root replacement (27.1% vs 12.7%, *P* = 0.03). In contrast, patients receiving aortic root replacement were more likely to suffer from prosthetic valve endocarditis (63.5% vs 24.7%, *P* < 0.001) and were more likely to need additional CABG (25.4% vs 11.8%, *P* = 0.03).

The mean EuroScore was 13.0 ± 7.8 points in the aortic root repair group and 15.4 ± 9.3 points in the aortic root replacement group (*P* = 0.08). Perioperative mortality was 18.8% (16 patients) in the aortic root repair group and 30.2% (19 patients) in the aortic root replacement group (*P* = 0.11).

Table 2 displays the extent of the disease in both treatment groups.

**Table 2:** Operative details of patients with destructive aortic root abscesses undergoing either aortic root repair or replacement

	Aortic root repair	Aortic root replacement	*P*-value
	*n* = 85 patients	*n* = 63 patients	
Additional abscess in, *n* (%)			
Left ventricular outflow tract	17 (20.0)	9 (14.3)	0.37
Intervalvular fibrous body	22 (25.9)	6 (9.5)	**0.01**
Mitral annulus	21 (24.7)	8 (12.7)	0.07
Perforation into, *n* (%)			
Left atrium	6 (7.1)	0 (0.0)	
Right atrium	7 (8.2)	5 (7.9)	
Right ventricle	3 (3.5)	2 (3.2)	0.19
Concomitant surgery, *n* (%)			
Mitral valve replacement	28 (32.9)	5 (7.9)	**<0.001**
Mitral valve repair	15 (17.6)	3 (4.8)	**<0.001**
Coronary artery bypass grafting	10 (11.8)	16 (25.4)	**0.03**
Aortic surgery	1 (1.2)	8 (12.7)	**0.005**
Tricuspid valve surgery	5 (5.9)	4 (6.3)	1.0
Root conduit used, *n* (%)			
Human homograft		5 (8.0)	
Biointegral conduit		3 (4.8)	
Freestyle porcine root		55 (87.3)	
Aortic prosthesis used, *n* (%)			
Mechanical	54 (63.5)		
Biological	31 (36.5)		
Cardiopulmonary bypass time (min), mean ± SD	197.6 ± 71.3	246.3 ± 79.2	**<0.001**
Aortic cross-clamp time (min), mean ± SD	123.8 ± 40.3	163.4 ± 43.9	**<0.001**
Extracorporeal membrane oxygenation after surgery, *n* (%)	3 (3.5)	12 (19.0)	**0.004**
Postoperative pacemaker implantation, *n* (%)	5 (5.9)	11 (17.5)	**0.03**

SD: standard deviation.

Patients receiving aortic root repair were more likely to have abscesses in the intervalvular fibrous body (25.9% vs 9.5%, *P* = 0.01) and the mitral annulus (24.7% vs 12.7%, *P* = 0.07). Bold emphasis means p<0.05.

Patients with aortic root repair were more likely to receive additional mitral valve replacement (32.9% vs 7.9%, *P* < 0.001) and mitral valve repair (17.6% vs 4.8%, *P* < 0.001) whereas patients with aortic root replacement were more likely to receive CABG (25.4% vs 11.8%, *P* = 0.03) and concomitant surgery of the ascending aorta (12.7% vs 1.2%, *P* = 0.005).

A Freestyle^®^ porcine root was used for aortic root replacement in the majority of cases (55 patients, 87.3%), a Biointegral^®^ conduit was implanted in 3 patients (4.8%) and a conventional cryopreserved homograft was taken as root substitute in 5 patients (8.0%). In the aortic root repair group, the majority of patients received a mechanical prosthesis for aortic valve replacement (54 patients, 63.5%).

CPB time (246.3 ± 79.2 vs 197.6 ± 71.3 min, *P* < 0.001) and aortic cross-clamp time (163.4 ± 43.9 vs 123.8 ± 40.3 min, *P* < 0.001) were significantly longer in the aortic root replacement group. In addition, more patients in the aortic root replacement group required insertion of an extracorporeal membrane oxygenation in the intra- or postoperative clinical course (19.0% vs 3.5%, *P* = 0.004). Moreover, there was a higher need for permanent pacemaker implantation for atrioventricular heart block in the aortic root replacement group (17.5% vs 5.9%, *P* = 0.03).

### Long-term outcome of patients with destructive aortic valve endocarditis

**Figure 1:**
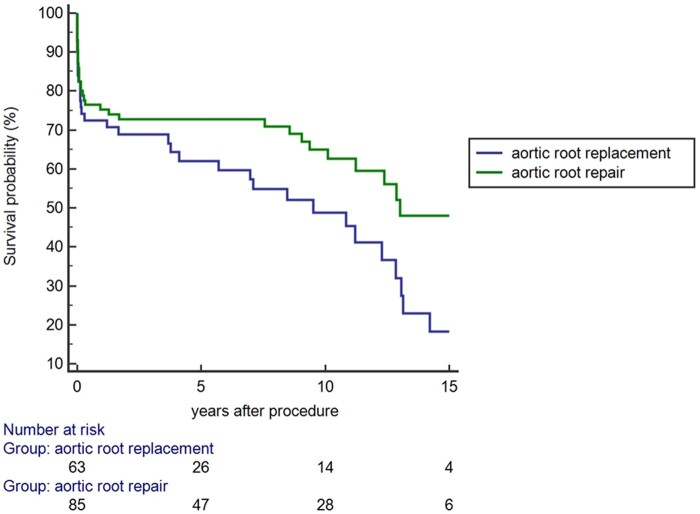
Long-term survival of patients receiving either aortic root repair (green line) or aortic root replacement (blue line). Long-term survival was significantly higher in the aortic root repair group (log-rank: *P* = 0.037).

Figure 1 displays the long-term survival of the entire cohort of patients. Patients receiving aortic root repair showed significantly better long-term survival compared to patients receiving aortic root replacement (log-rank: *P* = 0.037).

**Figure 2:**
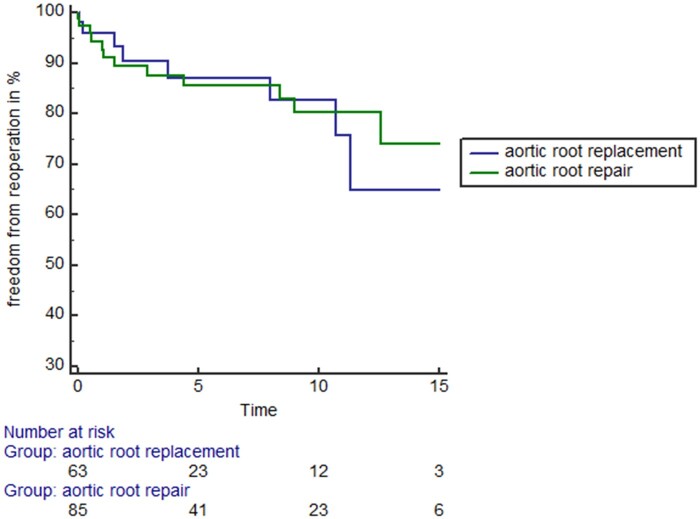
Long-term freedom from reoperation of patients receiving either aortic root repair (green line) or aortic root replacement (blue line). Long-term freedom from reoperation was not statistically different among both treatment groups (log-rank: *P* = 0.58).

Figure 2 shows the long-term freedom from valvular reoperation of the patient population. There was no statistical significant difference in terms of freedom from valvular reoperations among both treatment groups (log-rank: *P* = 0.58).

**Figure 3:**
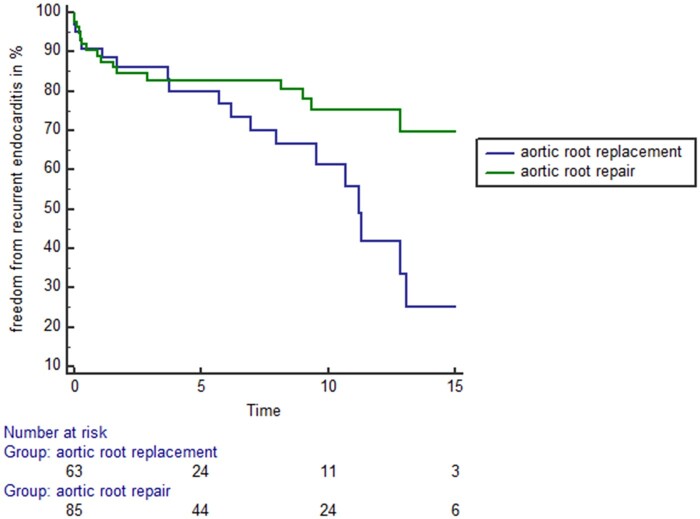
Long-term freedom from recurrent endocarditis of patients receiving either aortic root repair (green line) or aortic root replacement (blue line). Patient with aortic root repair showed significantly higher freedom from recurrent endocarditis compared to patients with aortic root replacement (log-rank: *P* = 0.022).

Figure 3 demonstrates the freedom from recurrent endocarditis among patients receiving either aortic root repair or replacement. Patients with aortic root repair showed significantly higher freedom from recurrent endocarditis compared to patients with aortic root replacement (log-rank: *P* = 0.022).

**Figure 4:**
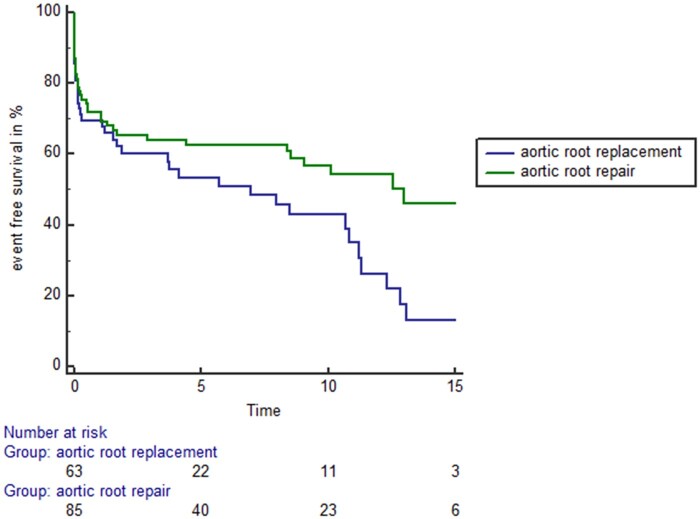
Event-free survival of patients receiving either aortic root repair (green line) or aortic root replacement (blue line). Patients with aortic root repair showed higher event-free survival compared to patients receiving aortic root replacement (log-rank: *P* = 0.022).

Figure 4 displays the event-free survival of the study cohort. Event-free survival was defined as a combination end point of freedom from death, valvular reoperation or recurrent endocarditis. Patients with aortic root repair showed higher event-free survival compared to patients receiving aortic root replacement (log-rank: *P* = 0.022).

Table 3 shows the result of the multi-variate Cox regression analysis. Neither native valve endocarditis (hazards ratio: 0.86, 95% confidence interval: 0.49–1.50; *P* = 0.59) nor double valve endocarditis (hazards ratio: 1.00, 95% confidence interval: 0.55–1.84; *P* = 0.99) was a statistically significant predictor for long-term event-free survival. Again, neither previous embolic stroke (hazards ratio: 1.22, 95% confidence interval: 0.74–2.02; *P* = 0.44) nor the need for additional CABG (hazards ratio: 1.34, 95% confidence interval: 0.73–2.46; *P* = 0.35) had influence on the long-term event-free survival. Increased age increased the risk of mortality by 2.1% per year. Aortic root repair showed significantly improved long-term event-free survival (hazards ratio: 0.59, 95% confidence interval: 0.35–0.98; *P* = 0.042) in the multi-variable adjusted statistical analysis. Urgency of operation—as described as the latency between beginning of antibiotic treatment and operation—was an independent predictor for inferior event-free survival.

**Table 3:** Results of the multivariable adjusted Cox regression analysis concerning event-free survival

	Hazards ratio	95% confidence interval	*P*-value
Age per year	1.021	1.004–1.039	**0.018**
Aortic root repair	**0.59**	**0.35–0.98**	**0.042**
Native valve endocarditis	0.86	0.49–1.50	0.59
Double valve endocarditis	1.002	0.55–1.84	0.99
Cerebral stroke prior to surgery	1.22	0.74–2.02	0.44
Additional CABG	1.34	0.73–2.46	0.35
Latency between beginning of antibiotic treatment and operation			
0–3 days	1		**0.001**
4–7 days	0.43	0.25–0.74	**0.002**
>7 days	0.41	0.24–0.72	**0.002**

Included variables were derived from univariate Kaplan–Meier survival analysis; variables analysed: age, gender, arterial hypertension, dyslipidaemia, obesity, peripheral arterial disease, chronic obstructive pulmonary disease, diabetes, previous bicuspid aortic valve, previous stroke, previous complicated stroke (meningitis, haemorrhage, abscess), peripheral embolism, latency between beginning of antibiotic treatment and operation, prosthetic valve endocarditis, additional CABG, aortic root repair, double valve endocarditis, postoperative pacemaker implantation, ECMO after surgery, impaired left ventricular function, previous cardiac decompensation, staphylococcal endocarditis, positive valve culture, additional aortic surgery, biological versus mechanical valve substitute, reoperation, and postoperative revision due to bleeding. Bold emphasis means p<0.05.

## DISCUSSION

Aortic root endocarditis remains a devastating condition with limited prognosis despite aggressive surgical treatment. The goal is a durable repair that is resistant to recurrent infection. The surgical repair technique remains a matter of discussion. The use of homografts has been postulated for many years, as the preservation treatment might favour resistance to reinfection. However, recent data suggest rapid degeneration and accelerated aortic valve dysfunction in homografts [9]. Aortic valves from homografts exhibited higher calcium scores, probably due to T-cell-mediated immunogenic response compared to xenografts [7].

Due to their broad availability and their favourable immunogenic properties, xenografts remain a valuable choice of prosthesis in root endocarditis. In a previous paper, our group has reported a series of 32 patients with severe destructive root endocarditis treated successfully with Freestyle root replacement [8]. Ten-year survival was 53% in this series. Similar results were reported from other groups validating the Freestyle prosthesis as valuable choice of treatment for root replacement in endocarditis [11].

However, as many affected patients are younger than 60 years, deterioration of the xenograft remains challenging. For such patients, the annular repair followed by implantation of a prosthesis might represent a valuable treatment option. In a series of 172 patients with root endocarditis, a 5-year survival rate of 50% was reported with recurrence of endocarditis in 9% of the patients [10]. Predictors of mortality included sepsis, concomitant CABG and prosthetic valve endocarditis.

Only few studies are available comparing the outcomes of different techniques for the surgical repair of aortic root endocarditis. In a study involving 150 patients, outcomes of patients with aortic root replacement with autologous or bovine pericardium or root repair with subsequent valve replacement were compared [12]. There was no difference in overall survival or freedom from reoperations between the groups. In a similar study, however, regarding reoperative root replacement, 130 patients with aortic root replacement (freestyle or mechanical composite) or isolated valve repair or replacement were investigated [13]. Ten-year survival was best in patients after valve-preserving root replacement. It remains unknown which prosthesis is superior in patients reoperated for prosthetic valve endocarditis. Reoperation for prosthetic valve endocarditis is challenging and outcomes impaired [10, 13].

Here, we report of a consecutive case series of 148 patients with destructive root endocarditis, 57% of whom were treated with aortic root repair (annular reconstruction with bovine pericardium followed by aortic valve replacement), whereas 43% received aortic root replacement (by means of a Freestyle xenograft prosthesis in the vast majority).

In conclusion, our data indicate that both aortic root repair and replacement are feasible options for the surgical treatment of root endocarditis. They represent complementary methods, depending on the extent of infection. Patients with less infection have a more favourable prognosis when receiving aortic root repair.

### Limitations

Our patient cohorts showed differences in their baseline characteristics. Patients receiving root replacement had more often prosthetic valve endocarditis, whereas the mitral valve was involved more often in the repair group. Patients receiving root replacement received more often a concomitant CABG or aortic surgery and exhibited increased CPB und X-clamp time. Moreover, there was more often a need for postoperative ECMO in patients with root replacement. However, beside age and urgency of operation, aortic root repair remained the single predictive factor in a multivariable adjusted analysis. Patients with root repair had higher freedom from recurrent endocarditis. Interestingly, there was no difference in freedom from reoperation between the groups. Patients receiving aortic root repair showed improved long-term survival and improved event-free survival, with a combined end point of freedom from death, valvular reoperation or recurrent endocarditis. Whether this might be due to lower deterioration rates in the repair group remains to be investigated in future trials.

Root endocarditis is an aggressive disease, and the extent of infection might be decisive for the surgical technique for the treatment of root endocarditis. Patients with advanced disease might more often receive root replacement rather than repair. Therefore, patients with a more advanced stage of the disease might be in the replacement group and thus show impaired prognosis. Thus, patients with root endocarditis might benefit from early surgery.

**Conflict of interest:** none declared. 

### Author contributions

**Can Gollmann-Tepeköylü:** Conceptualization; Data curation; Formal analysis; Investigation; Project administration; Writing—original draft. **Hannes Abfalterer:** Data curation; Writing—review & editing. **Leo Pölzl:** Data curation; Writing—review & editing. **Ludwig Müller:** Project administration; Supervision; Writing—review & editing. **Michael Grimm:** Formal analysis; Supervision; Writing—review & editing. **Johannes Holfeld:** Data curation; Methodology; Writing—review & editing. **Nikolaos Bonaros:** Conceptualization; Methodology; Supervision; Writing—review & editing. **Katie Bates:** Formal analysis; Investigation; Methodology; Software. Hanno Ulmer: Conceptualization; Formal analysis; Investigation; Methodology; Software. **Elfriede Ruttmann:** Conceptualization; Data curation; Formal analysis; Investigation; Methodology; Project administration; Supervision; Writing—original draft.

### Reviewer information

Interactive CardioVascular and Thoracic Surgery thanks Jan Vojacek and the other, anonymous reviewer(s) for their contribution to the peer review process of this article.

## Notes

Presented at the 34th Annual Meeting of the European Association for Cardio-Thoracic Surgery, Barcelona, Spain, 8–10 October 2020.

## ABBREVIATIONS

CPB: Cardiopulmonary bypass
IE: Infective endocarditis
LVOT: Left ventricular outflow tract
